# Using exploratory graph analysis (EGA) in validating the structure of the Perth alexithymia questionnaire in Iranians with chronic pain

**DOI:** 10.3389/fpsyg.2024.1400340

**Published:** 2024-07-03

**Authors:** Farzin Bagheri Sheykhangafshe, Hojjatollah Farahani, Peter Watson

**Affiliations:** ^1^Department of Psychology, Faculty of Humanities, Tarbiat Modares University, Tehran, Iran; ^2^MRC Cognition and Brain Sciences Unit, Cambridge University, Cambridge, United Kingdom

**Keywords:** exploratory graph analysis, perth alexithymia questionnaire, bootstrap, chronic pain, psychometric

## Abstract

**Background:**

Chronic pain’s influence on emotional well-being can be significant. It may evoke feelings of despair, frustration, nervousness, and melancholy in individuals, which often manifest as reactions to enduring pain and disruptions in their daily lives. In this study, we seek to perform Bootstrap Exploratory Graph Analysis (EGA) on the Persian Version of the Perth Alexithymia Questionnaire (PAQ) in a cohort of people with chronic pain.

**Methods:**

The research concentrated on the population of individuals encountering chronic pain within Tehran province from 2022 to 2023. Ultimately, the analysis comprised information from 234 male participants (with a mean age of 30.59, SD = 6.84) and 307 female participants (with a mean age of 30.16, SD = 6.65). After data collection, statistical analysis was conducted using the EGAnet2.0.4 package in R.4.3.2 software.

**Results:**

The outcome of bootstrapped EGA unveiled a two-dimensional configuration of the PAQ comprising Factor 1 denoted as negative difficulty in describing and identifying feelings (N-DDIF) and Factor 2 characterized as general-externally orientated thinking (GEOT), representing robust structural integrity and item consistency (all items have stabilities > 0.70).

**Conclusion:**

These findings endorse the validity of the PAQ, as evidenced by its confirmation in a broader sample using a novel methodology consistent with existing literature on two-factor decentering models.

## 1 Introduction

Chronic pain (CP) is a prevalent condition that impacts numerous individuals globally and significantly disrupts their daily lives (Clauw et al., 2019). The International Association for the Study of Pain (IASP) defines CP as an unpleasant sensory or emotional experience linked to actual or potential tissue damage. Pain can be either acute, lasting less than three months and often resulting from illness or injury, or chronic, persisting for at least three months, potentially involving tissue damage and recurring over time (Parolini et al., 2023).

The widespread nature of CP is evident across various countries. CP is experienced by 46% of individuals in Sweden (Jakobsson, 2010), 20.5% of people in the USA (Yong et al., 2022), 34.9% of people in Hong Kong (Wong and Fielding, 2011), 18.9% of people in Canada (Schopflocher et al., 2011), 35% to 51% of people in the UK (Fayaz et al., 2016), 23% to 76% of people in Brazil (Aguiar et al., 2021), 39.3% of people in Japan (Inoue et al., 2015) and 46.4% of people in Saudi Arabia (Almalki et al., 2019). Additionally, studies conducted in Iran reported a prevalence of 23.7% for CP (Shaygan et al., 2023).

CP is acknowledged as a multifaceted experience encompassing emotional and cognitive elements. It is understood to be a psychological-social phenomenon wherein biological, psychological, and social factors intertwine to shape the perception of pain (Pate et al., 2019). Alexithymia is one of the most influential psychological factors in the development of CP (Saariaho et al., 2016). Alexithymia’s importance in developing CP is characterized by difficulties in expressing emotions and identifying feelings (Fresán et al., 2021). The lack of emotional awareness inherent in alexithymia renders individuals more susceptible to stress, with CP often serving as a conduit through which emotions and stress-related sentiments manifest as physical symptoms (Lankes et al., 2020). Those with elevated levels of alexithymia are predisposed to negative experiences due to challenges in emotional differentiation and their somatic expressions (Torlak et al., 2022).

Patients grappling with CP encounter heightened difficulties in regulating emotion., These are often accompanied by associated physical ailments (La Touche et al., 2021). Research indicates that elevated levels of alexithymia are linked to various adverse outcomes in individuals with CP, including somatization (Lanzara et al., 2020), diminished quality of life (Tesio et al., 2018), psychological distress (Ghiggia et al., 2022), sexual dysfunction (Aboussouan et al., 2021), eating disorders (Fazia et al., 2024), increased pain intensity (Aaron et al., 2019b), pain catastrophizing (Shim et al., 2018), anger (Di Tella et al., 2018), and post-traumatic stress disorder (Fang and Chung, 2023).

Given the significant impact of alexithymia on both the physical and mental well-being of individuals living with CP, it is paramount to begin by exploring tools for the assessment of alexithymia (Becerra et al., 2021). In recent years, Preece et al. (2018) in Australia developed a questionnaire specifically addressing alexithymia. This questionnaire comprises 24 items and delineates into five subscales: negative difficulty identifying feelings (N-DIF), positive difficulty identifying feelings (P-DIF), negative difficulty describing feelings (N-DDF), positive difficulty describing feelings (P-DDF), and general externally oriented thinking (G-EOT). The validity and reliability of this questionnaire, known as the PAQ, have been examined across various countries. Larionow et al. (2022) conducted a study in Poland, assessing the psychometric properties of the PAQ in a Polish general community sample. Their findings indicated five factors having convergent and divergent validity with established psychological distress and mental health questionnaires.

Similarly, Chan et al. (2023) investigated the psychometric properties of the PAQ in both Australian and Singaporean populations revealing acceptable validity and reliability in both these cultures. Notably, higher scores were observed for both DIF and DDF in the Singaporean culture. Furthermore, Becerra et al. (2021) reported satisfactory validity and reliability of the PAQ in a Spanish sample. Despite these extensive validations, no research has yet assessed the psychometric properties of this questionnaire specifically in individuals grappling with CP. The current study, therefore, employs a novel statistical approach to evaluate the PAQ in patients dealing with CP, aiming to shed light on its utility and effectiveness in this specific population.

In previous research, factor-analytic approaches have been commonly utilized to assess the validity and reliability of various questionnaires (Koyuncu and Kilic, 2021). Confirmatory factor analysis, for example, aims to ascertain whether the number of factors and the loadings of measured variables on these factors align with theoretical models and frameworks (Cosemans et al., 2022). In recent years, however, a novel statistical method called Exploratory Graph Analysis (EGA) has emerged as a prominent tool in psychological research (Christensen and Golino, 2021). The introduction of the EGA package has transformed psychological research by offering user-friendly and freely available software for visualizing and estimating networks. Consequently, EGA has played a pivotal role in popularizing network methods in psychology (Koyuncu and Kilic, 2021).

The network perspective on psychological structures has given rise to a burgeoning subfield known as network psychometrics (Golino et al., 2022). Network models, typically utilizing Gaussian graphical models, estimate relationships among multiple variables by connecting nodes (e.g., test items) with edges representing the strength of relationships between variables (Van Der Maas et al., 2006). Golino and Epskamp (2017) demonstrated that a Gaussian graphical model combined with a clustering algorithm for weighted networks (such as Walktrap) could accurately recover the simulated number of factors, outperforming traditional factor analysis methods. This approach was termed EGA by Golino and Epskamp (2017). Building on this foundation, Golino et al. (2020) conducted further research comparing EGA with various types of factor analytic methods, including two types of parallel analysis.

This study endeavors to fill this research void by employing EGA to assess the psychometric characteristics of the PAQ in individuals coping with CP. Through the application of this pioneering statistical methodology, we aim to deepen our comprehension of alexithymia within the realm of CP and to guide the development of targeted clinical interventions.

## 2 Materials and method

### 2.1 Participants

This study focused on individuals experiencing CP in Tehran province between 2022 and 2023. From a total of 600 participants who completed the research questionnaires, 59 were excluded due to inconsistencies or distortions in their responses. This left 234 males (mean age = 30.59, SD = 6.84) and 307 females (mean age = 30.16, SD = 6.65) in the sample used for analysis. The inclusion criteria for the study encompassed personal satisfaction, a history of experiencing CP within the past two years, the absence of mental disorders, and no current receipt of psychological counseling services. Conversely, exclusion criteria among participants consisted of incomplete questionnaire submissions and instances of a worsening of the disease.

### 2.2 Measures

#### 2.2.1 Demographic information checklist

The researcher-designed checklist comprised details regarding participants’ age, gender, marital status, level of education, duration of CP, and specific type of CP experienced.

#### 2.2.2 Perth Alexithymia Questionnaire (PAQ)

Perth Alexithymia Questionnaire developed by Preece et al. in 2018, consists of a self-report form comprising 24 items and 5 subscales. It aims to evaluate difficulties in identifying and expressing emotions. and to pinpoint external thought elements associated with alexithymia. Respondents rate each item on a 7-point Likert scale ranging from 1 (strongly disagree) to 7 (strongly agree), yielding total scores between 24 and 168. Higher scores indicate greater challenges in identifying individual emotions. The subscales include difficulty identifying feelings associated with negative emotions (items 2, 8, 14, 20), difficulty identifying feelings associated with pleasant emotions (items 5, 11, 17, 23), difficulty describing feelings associated with negative emotions (items 1, 7, 13, 19), difficulty describing feelings associated with pleasant emotions (items 4, 10, 16, 22), and general-externally orientated thinking (items 3, 6, 9, 12, 15, 18, 21, 24). The validity of the questionnaire was established through concurrent validity analysis, demonstrating that individuals reporting higher levels of alexithymia also exhibited greater difficulties in the regulation of emotion and higher levels of psychological distress. Concurrently, the internal consistency (reliability) of the questionnaire was assessed using Cronbach’s alpha resulting in very good values ranging from 0.89 to 0.91 for all subscales (Preece et al., 2018). In the current study, favorable Cronbach’s alpha coefficients were reported for both the total score (0.88) and the subscales of N-DIF (0.85), P-DIF (0.83), N-DDF (0.85), P-DDF (0.85) and GEOT (0.88).

### 2.3 Procedures

Following receipt of the ethics code from the Ethics Committee of Tarbiat Modares University (IR.MODARES.REC.1401.197), a roster of relevant centers was compiled, and initial coordination ensued with the respective authorities. Subsequently, 600 individuals with chronic pain were purposefully selected, and the research objectives were elucidated to them. Upon obtaining consent from their attending physician, participants were tasked with accurately completing research questionnaires and answering inquiries to the best of their ability. Each participant dedicated approximately 15 to 20 minutes to complete the research questionnaires. Ultimately, 541 individuals with chronic pain actively engaged in the study. Data acquired from questionnaire responses were analyzed employing SPSS v24 and R. The significance level for this investigation was established at 0.05.

### 2.4 Data analysis

To investigate the number of dimensions of the PAQ-24 scale exploratory graph analysis (Golino et al., 2020) was performed using the EGAnet2.0.4 package (Hudson and Alexander, 2022) in R.4.3.2 software (R Core Team., 2022). The GLASSO method was used to estimate the coefficients in the graph. This method is a penalized maximum likelihood solution based on the extended Bayesian information criterion (EBIC, Chen and Chen, 2008; Epskamp et al., 2012).

Exploratory graph analysis (EGA) provides a framework for measuring dimensionality. EGA determines the number of dimensions in psychological data using network estimation methods and cluster discovery algorithms. The bootstraps method is used to examine the stability of dimensions and items. EGA was done in several steps in this research. In the first step, redundancy analysis is performed on the items of a scale. With this method items with high correlation are identified by looking at the effects of wording in the writing of the items. Redundant items can hurt the estimation of the dimensions of the investigated scale therefore they should be checked and removed first. In EGA, unlike classical factor analysis, in which it is assumed that the items of a scale measure latent common variables, it is assumed that in EGA the network of items is causally autonomous (Christensen et al., 2020a,b).

The graphical least absolute shrinkage and selection operator (GLASSO) was used in the EGA. GLASSO measures the matrix of discriminant correlations (Regularized partial correlations) in which the matrix nodes are the items and the edges are the partial correlations between the items. Algorithms can be used to find the number of dimensions. The walktrap algorithm was used in this analysis. This algorithm detects the number of factors (dimensions) that have items that strongly correlate with each other (Golino and Epskamp, 2017).

We used bootstrapping with 1000 iterations to remove the bias effect caused by sampling variability in EGA and so make the results more robust (Christensen and Golino, 2021; Farahani et al., 2023).

In the next step, we checked the reliability and stability of the items and the structural stability of the network. The stability of the extracted dimensions in all the samples resulting from the bootstrapping method was assessed by computing in how many of the 1000 bootstrap samples an item appeared on each dimension. Christensen and Golino (2021) recommend that if the item stability index for an item on a scale is low (< 0.7), it should be removed. In this research, these items were removed and the analysis was done again. Cronbach’s alpha and McDonald’s omega were also used to assess the reliability of this scale.

Confirmatory factor analysis (CFA) was used to check construct validity. The estimation method in this analysis was maximum likelihood. Absolute and relative fit indices were used to check the fit of the model. RMSEA, SRMR, and CFI were used for absolute fit and x^2^/df was used since the chi-square value (x^2^) is sensitive to the sample size (Kline, 2023)., A good fit has RMSEA and SRMR less than 0.05 and x^2^/df less than 2 (Cangur and Ercan, 2015), CFI is a comparative fit index of the model. If its value is higher than 0.95 the model fit is excellent (Jackson et al., 2009). AIC indices can also be used to check the relative fit of the models. In EGA, the so-called Total Entropy Fit index (TEFI) is also used, which shows how much uncertainty there is. The lower the value, the stronger the factor structure (Golino et al., 2021). In this research, all the above indicators were calculated and reported. The Lavaan package (Rosseel, 2012) was used to perform confirmatory factor analysis with DWLS estimation which is robust to non-normality in the items.

## 3 Results

The age range of patients with CP was 18 to 45 years. A total 307 participants were female (43.3%), and 234 were male (56.7%). 253 participants were single (46.8%) and 288 (53.2%) were married. Participants had varying educational backgrounds, with 57 (10.5%) having a Diploma, 152 (28.1%) holding an associate degree, 157 (29.0%) with a Bachelor’s degree, 126 (23.3%) with a Master’s degree and 49 (9.1%) holding a Ph.D. Participants reported durations of chronic pain ranging from 2 to 9 years. The highest number of participants reported experiencing chronic pain for 2 years (*N* = 150, 27.7%). Participants reported experiencing various types of chronic pain, including Backache (*N* = 115, 21.3%), Neck Pain (*N* = 191, 35.3%), Headache (*N* = 90, 16.6%) Fibromyalgia (*N* = 62, 11.5%), Rheumatoid Arthritis (*N* = 51, 9.4%), and Pelvic pain (*N* = 32, 5.9%). Table 1 gives the demographic information of the participants.

**TABLE 1:** Demographic information of participants.

		*n*	Percent
Gender	Female	307	43.3
	Male	234	56.7
Marital status	Single	253	46.8
	Married	288	53.2
Age	18 to 26	187	34.5
	27 to 36	248	45.9
	37 to 45	106	19.6
Education	Diploma	57	10.5
	Associate degree	152	28.1
	Bachelor	157	29.0
	Masters	126	23.3
	Ph.D.	49	9.1
Duration of chronic pain	2	150	27.7
	3	108	20.0
	4	87	16.1
	5	83	15.3
	6	63	11.6
	7	15	2.8
	8	23	4.3
	9	12	2.2
Types of chronic pain	Backache	115	21.3
	Neck Pain	191	35.3
	Headache	90	16.6
	Fibromyalgia	62	11.5
	Rheumatoid Arthritis	51	9.4
	Pelvic pain	32	5.9
Total		541	100.00

Table 2 shows the results of confirmatory factor analysis (CFA) on 24 items of the PAQ scale along with the results of factor analysis on 13 items after the implementation of EGA and the removal of unstable items (with stability less than 0.7). As the results of Table 2 show, the main 5-factor structure of the scale had a good fit. (*x*^2^(242) = 194.57  *p* < 0.001, *x*^2^/*df* = 3.042, *RMSEA* = 0.051, *SRMR* = 0.034, *CFI* = 0.97)

**TABLE 2:** Model fit indices of different factors of the PAQ.

Model	x^2^	df	x^2^/df	P	CFI	RMSEA	SRMR	AIC	TEFI
CFA	746.71	234	3.08	0.001	0.935	0.062	0.038	41548.06	–
EGA	194.57	64	3.04	0.001	0.971	0.051	0.034	23106.62	−563.16

CFA, original structure (5-factor); EGA, CFA after running EGA and omitting Item 23, item5, item 15, item 2, item4, item10, item22, item 11, item 17, item16, and item 8.

**FIGURE 1:**
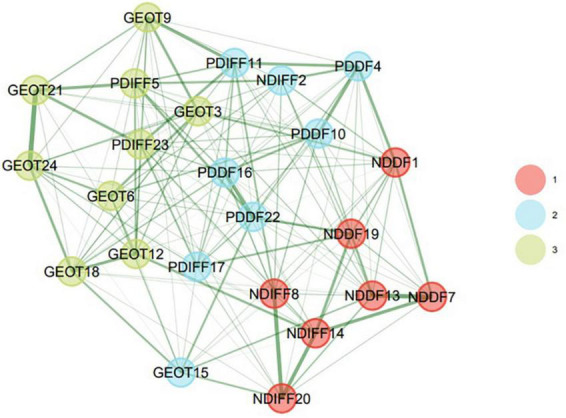
Typical network structure for the 24 items of the PAQ with regulated edge weights for its four communities based on median partial correlations over 10,000 bootstraps.

The fit indices confirm the main 5-factor structure of the scale and all the items in their respective factors (dimensions) have a significant standardized factor loading above 0.3. The factor structure of the PAQ scale was also examined based on the results of the EGA. First, redundancy analysis using Unique Variance Analysis (UVA) was used to check the items. The results showed that there was no significant local dependency and no item had a correlation higher than 0.19, which indicates low location dependency (Rader et al., 2023) therefore, no items were removed at this stage. Figure 1 gives the network resulting from EGA with GLASSO estimation., The network has 24 nodes and 159 edges with an edge density of 0.576. The mean, standard deviation, min, and max for non-zero edge weights are respectively 0.07, 0.054, 0.002, and 0.268 (Figure 1).

Table 3 summarizes the bootstrapping and shows that the median number of dimensions is 3 with a 95% confidence interval between 2 and 5 dimensions. Table 4 shows the frequency of PAQ dimensions with and without the removal of unstable items after bootstrapping.

**TABLE 3:** Summary of non-parametric Bootstrapping.

N.Boots	Median.dim	SE.dim	CI.dim lower	CI.dim upper
1000	3	0.89	1.76	4.76

**TABLE 4:** Frequencies of the number of PAQ dimensions across all replicate samples.

	Proportion of replicates with given number of factors including unstable items		Proportion of replicates with given number of factors without unstable items
1	0.082	1	0.004
2	0.194	2	0.996
3	0.559		
4	0.137		
5	0.037		

**FIGURE 2:**
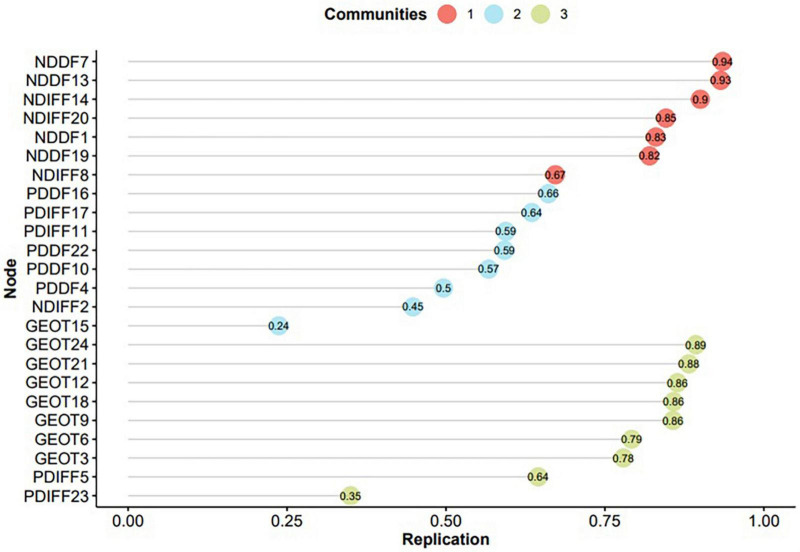
Item stability plot of the PAQ-24 based on bootstrapped EGA results.

As shown in Table 4 the three-factor structure appeared in most bootstrap samples being present in 55.9% of the iterations. Figure 2 shows the stability of items after bootstrapping. The stability values of the items fluctuate from 0.24 for item 15 to 0.94 for item 7. According to the recommendation of the researchers, items with low stability (below 0.7) should be removed, which in this study included items 23, 5, 15, 2, 4, 10, 22, 11, 17, 16, and 8, which were, therefore, removed and the EGA model refitted.

**FIGURE 3:**
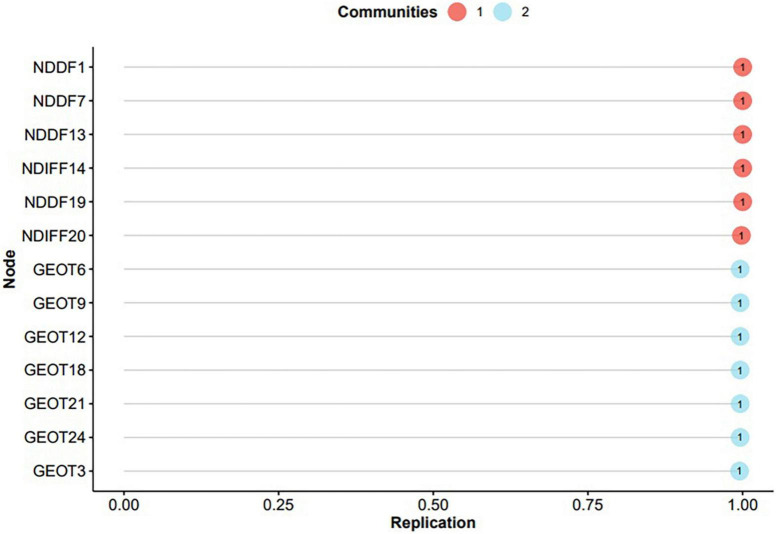
Item stability plot of the PAQ based on bootstrapped EGA results (excl. 11 unstable items).

Using an EGA with GLASSO gave 13 items linked by 58 edges, with an edge density of 0.744. The mean, standard deviation, min, and max respectively for the non-zero edge weights were 0.102, 0.072, 0.001, and 0.302. The percentage of bootstrap samples featuring different numbers of factors can be seen in the right part of Table 4. This suggests a two-factor structure. Checking the stability of the items showed that all the items resulting from this stage have full stability and were equal to 1 in two dimensions (Figure 3).

Table 5 shows the structural stability of the factors obtained from EGA with all the items and after removing the unstable items.

**TABLE 5:** Structural stability.

Factor	EGA (total)	EGA (without unstable items)
1	0.522	0.998
2	0.092	0.995
3	0.230	–

Confirmatory factor analysis was performed based on the results of the EGA. Table 6 shows the standardized loadings, t statistics, and p-values associated with the model with two factors (PAQ1 and PAQ2).

**TABLE 6:** Standardized loadings, *t* and *p* of items.

Factor	Items	ß	t	P
PAQ1	N-DDF1	0.752	–	0.001
	N-DDF7	0.750	17.345	0.001
	N-DDF13	0.748	17.288	0.001
	N-DIFF14	0.760	17.604	0.001
	N-DDF19	0.761	17.626	0.001
	N-DIFF20	0.675	15.490	0.001
PAQ2	GEOT3	0.744	–	0.001
	GEOT6	0.673	15.313	0.001
	GEOT9	0.723	16.516	0.001
	GEOT12	0.729	16.680	0.001
	GEOT18	0.728	16.641	0.001
	GEOT21	0.673	15.314	0.001
	GEOT24	0.755	17.313	0.001

**FIGURE 4:**
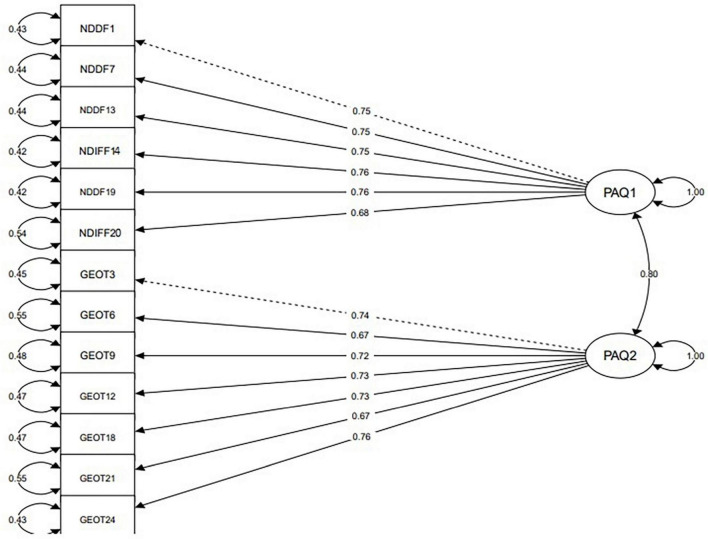
CFA plot for 2- factor structure of PAQ-13.

As Table 6 shows, all standardized loadings are above 0.3 and are meaningful (Figure 4).

Looking at the factor loadings these two factors from the PAQ scale were named as describing and identifying feelings associated with negative emotions (N-DDIF) and general-externally orientated thinking (GEOT). Table 3 (EGA row) indicates the fit indices of the 2- 2-factor structure of PAQ-13. N-DDIF and GEOT both have high correlations with PAQ-13 (Table 7).

**TABLE 7:** Descriptive statistics, Pearson, and unregulated partial correlations for two PAQ primary subtests.

	Variables	N-DDIF	G-EOT	PAQ Total
1	N-DDIF	1		
2	G-EOT	0.701**	1	
3	PAQ Total	0.913**	0.931**	1
4	M	21.81	23.45	45.26
5	SD	7.60	8.46	14.82
6	Skewness	0.147	0.080	0.243
7	Kurtosis	0.632	0.920	0.855
	*P<0.05 **P<0.01			

The findings indicated a positive and significant association between the PAQ and N-DDIF (*r* = 0.913, *p* < 0.001), and G-EOT (*r* = 0.931, *p* < 0.001). *p < 0.05; **p < 0.01.

### Reliability

The two-factor structure obtained from the bootstrapped EGA showed high structural stability and this structure was replicated in 99.6% of bootstrapped samples. The stability of items in this two-factor structure was excellent and below 0.7 for all the remaining items. Cronbach’s alpha coefficients, McDonald’s Omega, composite reliability (CR) and average variance extracted (AVE) for the two-factor model were very good. Table 8 indicates the reliability characteristics of PAQ-13.

**TABLE 8:** Reliability characteristics of the two-factor structure.

Factor	CR	Omega	Alpha	AVE
1	0.880	0.870	0.870	0.55
2	0.881	0.890	0.880	0.52
total	0.930	0.870	0.881	0.54

## 4 Discussion

The purpose of this article was to validate the factor structure of the Alexithymia scale. For this purpose, the five-factor structure of the original PAQ-24 was examined, and then the two-factor structure resulting from the EGA was also examined. The results of the confirmatory factor analysis confirmed the 5-factor structure proposed by Preece et al. (2020) with an acceptable fit to a community sample of Iranians with CP. When unstable items were removed the EGA on the total sample (*n* = 541) showed a 2-factor structure. Bootstrapping indicated the stability of the items in the two-factor structure of this scale, with the two factors defined as difficulty describing and identifying feelings associated with negative emotions (N-DDF and N-DIFF) and general-externally orientated thinking (GEOT). In EGA, items are considered causally autonomous. EGA, unlike classical factor analysis, does not assume that all items measure the same latent construct. Following Christensen et al. (2023) EGA performs redundancy analysis to eliminate items that are strongly correlated and have the possibility of being redundant.

Redundancy is often due to shared error variance which indicates that items may be very similar in terms of wording. The advantage of this research, therefore, is that items with local dependency are eliminated, yielding a stable structure as assessed by the use of the bootstrap. The items that show little stability (below 0.7) are removed leaving factors that show structural stability over the 1000 bootstrap repetitions.

Santiago et al., 2021, Laskowski et al., 2023, and Rader et al., 2023 emphasized these advantages and the robustness of the results in examining the psychometric properties of psychological scales with these methods. Alexithymia, a cognitive-affective trait, denotes an individual’s incapacity to properly recognize, experience, and articulate emotions (Lankes et al., 2020). This condition can significantly impact the mental well-being of individuals grappling with CP. The incapacity to acknowledge, undergo, and articulate emotions may result in diminished contentment with life, heightened stress and anxiety levels, and challenges in interpersonal connections (Fresán et al., 2021). Misidentified or unexpressed emotions can exacerbate pain, diminish the efficacy of pain management strategies, and lead to further physical and psychological complexities (Torlak et al., 2022). Furthermore, difficulties in emotional expression and empathizing with others’ viewpoints may diminish social interactions and foster sentiments of isolation and solitude in patients (Saariaho et al., 2016). Hence, grasping and addressing alexithymia is integral in therapeutic interventions for individuals enduring CP (Pate et al., 2019).

The presence of N-DDIF among individuals with CP presents a significant and intricate challenge in the realms of mental health and pain management (Aaron et al., 2019a). Those grappling with CP often endure a spectrum of negative emotions, including anxiety, depression, irritability, and distress, for various reasons (Pei et al., 2023) however, their ability to recognize and articulate these emotions may be constrained, leading to difficulties in expressing their feelings. This limitation can have multifaceted repercussions (Becerra et al., 2021) such as struggles in identifying and articulating negative emotions that may foster heightened self-disgust, increased levels of anxiety and depression, and a diminished sense of self-efficacy in managing pain (Chan et al., 2023). Furthermore, these challenges may impact the patient’s rapport with healthcare providers and mental health professionals hindering the provision of appropriate pain treatment and management (Preece et al., 2024).

GEOT, as a cognitive pattern, can compound the difficulties experienced by patients with CP (Diotaiuti et al., 2021). This cognitive style often leads to a fixation on negative and unstable beliefs and ideas, which can detrimentally affect the perception and handling of pain (Torlak et al., 2022). Individuals contending with CP may grapple with negative thoughts related to their pain experience, such as feelings of hopelessness, dissatisfaction, and mistrust, stemming from the persistent nature of their pain (Di Tella et al., 2018). GEOT can engender negative thought patterns in patients and impede the adoption of effective pain management strategies (Lankes et al., 2020). Consequently, patients may not fully derive benefits from various treatments or engage in psychological counseling and support (Aaron et al., 2019b). To enhance the management of CP in individuals, it is imperative to identify and address negative thought patterns associated with pain (Becerra et al., 2021). This may entail psychological counseling, stress management, skill training, and the implementation of positive thinking techniques (Pei et al., 2023). Heightening patients’ awareness of how to channel their thoughts towards positive states and employ effective pain management strategies can contribute to an enhanced quality of life and more effective CP management (Torunsky et al., 2023).

According to the research conducted thus far, there has been limited investigation into the correlation of the PAQ among patients with CP, with most studies opting for the Toronto Alexithymia Scale (TAS-20). In their 2024 study, Preece et al. aimed to assess the discriminant validity of the TAS-20 and the PAQ through exploratory factor analysis (EFA). The EFA identified two main factors: an “alexithymia” factor and a “general distress” factor, encompassing depression, anxiety, and stress. PAQ scores consistently loaded onto the alexithymia factor without cross-loading onto the distress factor. However, TAS-20 scores, particularly from the DIF facet, showed significant cross-loadings on the distress factor, prompting concerns regarding its discriminant validity. Larionow et al. (2024) conducted a validation study of the Russian version of the Perth Alexithymia Questionnaire-Short Form (PAQ-S), a concise self-report assessment for alexithymia. Confirmatory factor analysis affirmed the intended 1-factor structure of the PAQ-S indicating robust factorial validity. The PAQ-S demonstrated favorable internal consistency with good reliability and exhibited expected associations with symptoms of psychopathology and well-being, supporting its convergent and divergent validity. PAQ-S scores were predictive of diminished well-being underscoring the clinical significance of alexithymia as measured by the PAQ-S. Furthermore, no gender disparities in PAQ-S scores were observed, although alexithymia was more prevalent among younger individuals and those with lower levels of education.

Elevated levels of alexithymia often coincide with experiential avoidance, which can potentially worsen psychological distress however the specific mechanisms linking experiential avoidance to alexithymia and psychological distress at a latent construct level remain unclear. To investigate this, Torunsky et al. (2023) administered questionnaires to 693 U.S. adults, evaluating alexithymia, general distress, multi-dimensional experiential avoidance, and general health. Structural equation modeling revealed that alexithymia significantly predicted experiential avoidance which subsequently predicted general distress. Experiential avoidance fully mediated the relationship between alexithymia and general distress. Notably, correlations between alexithymia and subfactors of experiential avoidance emphasized a strong association with repression and denial. In their 2023 study, Pei et al. aimed to ascertain the prevalence of alexithymia among Chinese people with CP, employing the TAS-20 assessment. From an initial screening of 346 patients 321 with CP were included giving an alexithymia prevalence of 19.6%. The research identified anxiety, pain catastrophizing, and self-efficacy as autonomous predictors of alexithymia within this patient cohort. In their 2019 study, Aaron, Fisher, & Palermo sought to examine the prevalence of alexithymia in adolescents with CP in comparison to those without CP and to explore the correlation between alexithymia and pain experiences in youth. Their findings revealed that adolescents with CP displayed elevated total alexithymia scores, particularly showing difficulty identifying feelings, even after adjusting for psychological distress. Additionally, the study observed a significant correlation between difficulty identifying feelings and heightened levels of pain interference and annoyance due to pain.

### 4.1 Practical implications

Identifying specific facets of alexithymia, such as N-DDIF and GEOT, enables healthcare professionals to more accurately gauge emotional distress in chronic pain patients. This deeper comprehension empowers tailored interventions to target individual emotional hurdles. Furthermore, understanding the intricate emotional landscape linked to chronic pain facilitates the development of more efficient treatment approaches. By addressing both internal emotional processing challenges and external cognitive patterns, healthcare providers can deliver comprehensive care aimed at enhancing patient well-being. Mental health practitioners can use the validated PAQ to craft customized psychological support programs, aiding chronic pain patients in navigating their emotional journeys and enhancing their overall quality of life. Additionally, the utilization of Bootstrap EGA introduces a novel methodological paradigm for analyzing intricate datasets in psychological research. This advancement opens up new avenues for exploring and comprehending complex relationships between variables across diverse fields such as behavioral sciences, social sciences, life sciences, and bioinformatics. Ultimately, the insights garnered from this study contribute to the advancement of more effective assessment tools and treatment strategies for chronic pain patients, with the overarching goal of alleviating their emotional distress and enhancing their overall well-being.

Reducing the number of questions in a survey is crucial for improving the efficiency and effectiveness of data collection instruments. EGA offers a robust, data-driven approach to this process, ensuring essential constructs are measured effectively while making the survey more user-friendly and efficient. A key strength of EGA lies in its ability to identify the most representative items within each construct or cluster. By calculating centrality measures—such as strength, closeness, and betweenness—researchers can pinpoint items that are most central to their respective clusters. These central items are crucial because they capture core aspects of the construct, ensuring the survey remains comprehensive and accurate despite having fewer questions. For instance, in a psychological survey assessing anxiety, depression, and stress, centrality measures can help identify which items best represent each construct, allowing for a reduction in the total number of questions without losing essential information.

Redundancy analysis further enhances survey efficiency by eliminating items that provide overlapping information. High inter-item correlations within a cluster indicate redundancy, meaning some items do not add unique value. By removing these redundant items, the survey becomes more streamlined, reducing respondent burden and potential fatigue. This not only improves response rates but also enhances data quality, as respondents are less likely to disengage or provide inaccurate answers due to the survey’s length. Maintaining the psychometric integrity of the instrument is a major concern when reducing the number of survey questions. EGA, combined with redundancy analysis, ensures the reduced survey retains its reliability and validity. Reliability analysis, such as calculating Cronbach’s alpha, confirms the internal consistency of the constructs is maintained with the reduced item set. Additionally, validity testing, including confirmatory factor analysis (CFA), ensures the reduced survey still accurately reflects the theoretical constructs it aims to measure. These steps are crucial in ensuring the survey remains a reliable and valid tool for data collection.

The practical benefits of reducing the number of survey questions are significant. Shorter surveys are quicker to administer and complete, making them more practical in various settings, from clinical assessments to educational evaluations and market research. They also enhance the user experience by reducing respondent burden, leading to higher completion rates and more accurate data. Moreover, shorter surveys are cost-effective, reducing the expenses associated with data collection, processing, and analysis. These benefits make the reduced survey a more efficient tool for both researchers and respondents.

Using EGA to reduce the number of survey questions in the PAQ, as here to 13 items, is a methodologically sound and practically beneficial approach. By using centrality and redundancy analyses, researchers can retain the most representative items, ensuring essential constructs are measured accurately with fewer questions. This approach enhances the efficiency and user-friendliness of the survey, leading to higher response rates and better data quality. Additionally, maintaining the psychometric integrity of the survey through rigorous reliability and validity testing ensures the reduced instrument remains a robust and reliable tool for data collection.

The iterative process of validation and refinement, including pilot testing and adjustments based on feedback, further ensures the reduced survey performs well in real-world conditions. This comprehensive approach to item reduction makes EGA an invaluable tool in various research and applied settings, providing a balanced solution that optimizes both the efficiency and accuracy of survey instruments. In summary, the application of EGA for item reduction is a powerful strategy that enhances the practicality and reliability of surveys. By focusing on centrality and redundancy, and validating the reduced instrument, researchers can create concise, effective surveys that retain their psychometric properties, ultimately leading to more efficient and user-friendly data collection tools.

### 4.2 Limitations and future work

The study’s sample size, while considerable, was largely drawn from Tehran province, potentially restricting the applicability of the results to broader populations. Furthermore, the demographic distribution skewed towards younger age groups, necessitating caution in extrapolating findings to older cohorts. The cross-sectional nature of the study impedes the ability to establish causality between alexithymia dimensions and chronic pain outcomes. Longitudinal investigations are necessary to unravel the temporal dynamics of these associations over time. Relying solely on self-report measures, such as the PAQ, introduces the risk of response biases and social desirability effects. Future research could incorporate diverse assessment methods, including clinician-administered evaluations, to bolster the credibility of the findings. While Bootstrap EGA presents a robust analytical framework, alternative methodologies could offer supplementary insights into the underlying structure of alexithymia in chronic pain populations. Exploring diverse statistical techniques has the potential to deepen our understanding of this multifaceted phenomenon.

Future studies should utilize longitudinal designs to investigate the temporal relationships among alexithymia dimensions, chronic pain severity, and emotional well-being over extended durations. Longitudinal data have the potential to elucidate the trajectory of alexithymia symptoms and their impact on pain outcomes. Broadening the scope of research to encompass diverse geographical regions and demographic profiles can bolster the generalizability of the findings. Enrolling participants from varied cultural backgrounds and age groups can shed light on the universality of alexithymia constructs across populations. It is crucial to examine the effectiveness of tailored interventions targeting specific alexithymia dimensions in chronic pain management. Subsequent research endeavors should assess the efficacy of psychological interventions, such as emotion-focused therapy, in alleviating alexithymia-related distress and enhancing pain-related outcomes.

Exploring advanced analytical techniques, such as network analysis or machine learning algorithms, holds promise for gaining deeper insights into the interconnectedness of alexithymia dimensions and their predictive value for chronic pain outcomes.

Integrating multidimensional data sources can provide a more comprehensive understanding of the intricate interplay between psychological and physiological factors in chronic pain. Including individuals with comorbid psychiatric and medical conditions, such as depression, anxiety, and various chronic illnesses, would offer a more comprehensive view of the interaction between alexithymia and CP. By addressing these limitations and embarking on future avenues of research, researchers can advance our comprehension of alexithymia in the context of chronic pain and devise more effective interventions to enhance patient outcomes.

## 5 Conclusion

These findings endorse the validity of the PAQ, as evidenced by its confirmation in a broader sample using a novel methodology consistent with existing literature on two-factor decentering models. EGA is a statistical technique used to explore hidden patterns and intricate connections among variables within a network or graph by scrutinizing their interactions. This method is particularly potent for analyzing intricate and interlinked data, notably in domains such as behavioral sciences, social sciences, life sciences, and bioinformatics. In EGA, variables are depicted as nodes or vertices, with their interrelations portrayed as links or edges in a graphical representation. Through this approach, complex and implicit patterns of relationships among variables can be discerned. EGA serves as a valuable tool in theoretical and applied research for unveiling concealed patterns and elucidating associations between variables.

## Data availability statement

The raw data supporting the conclusions of this article will be made available by the authors, without undue reservation.

## Ethics statement

The studies involving humans were approved by the Tarbiat Modares University ethics committee. The studies were conducted in accordance with the local legislation and institutional requirements. The participants provided their written informed consent to participate in this study.

## Author contributions

FS: Conceptualization, Formal analysis, Writing−original draft. HF: Conceptualization, Project administration, Supervision, Writing−original draft. PW: Project administration, Writing−review and editing.
